# Risk factors for type 1 diabetes, including environmental, behavioural and gut microbial factors: a case–control study

**DOI:** 10.1038/s41598-020-74678-6

**Published:** 2020-10-16

**Authors:** Deborah Traversi, Ivana Rabbone, Giacomo Scaioli, Camilla Vallini, Giulia Carletto, Irene Racca, Ugo Ala, Marilena Durazzo, Alessandro Collo, Arianna Ferro, Deborah Carrera, Silvia Savastio, Francesco Cadario, Roberta Siliquini, Franco Cerutti

**Affiliations:** 1 Department of Public Health and Pediatrics, University of Turin, Piazza Polonia 94, 10126 Torino, Italy; 2 S.S.V.D. Endocrinology and Diabetology, O.I.R.M., Azienda Ospedaliera Città Della Salute E Della Scienza, Turin, Italy; 3 Paediatric Endocrinology, Azienda Ospedaliero Universitaria Maggiore Della Carità - Novara, Novara, Italy; 4 S.C.U. Medicina Interna 3, Azienda Ospedaliera Città Della Salute e Della Scienza Di Torino, Torino, Italy; 5 Department of Veterinary Sciences, University of Turin, Torino, Italy; 6 Dietetic and Clinical Nutrition Department, Azienda Ospedaliero Universitaria Maggiore Della Carità, Novara, Italy; 7 Department of Health Science, University of Eastern Piedmont Amadeo Avogadro - Azienda Ospedaliero Universitaria Maggiore Della Carità - Novara, Novara, Italy; 8 Department of Public Health and Pediatrics, Hygiene Unit, University of the Study of Turin, via Santena 5 bis, 10126 Torino, Italy

**Keywords:** Microbiology, Molecular biology, Biomarkers, Diseases, Risk factors

## Abstract

Type 1 diabetes (T1D) is a common autoimmune disease that is characterized by insufficient insulin production. The onset of T1D is the result of gene-environment interactions. Sociodemographic and behavioural factors may contribute to T1D, and the gut microbiota is proposed to be a driving factor of T1D. An integrated preventive strategy for T1D is not available at present. This case–control study attempted to estimate the exposure linked to T1D to identify significant risk factors for healthy children. Forty children with T1D and 56 healthy controls were included in this study. Anthropometric, socio-economic, nutritional, behavioural, and clinical data were collected. Faecal bacteria were investigated by molecular methods. The findings showed, in multivariable model, that the risk factors for T1D include higher Firmicutes levels (OR 7.30; IC 2.26–23.54) and higher carbohydrate intake (OR 1.03; IC 1.01–1.05), whereas having a greater amount of *Bifidobacterium* in the gut (OR 0.13; IC 0.05 – 0.34) was a protective factor for T1D. These findings may facilitate the development of preventive strategies for T1D, such as performing genetic screening, characterizing the gut microbiota, and managing nutritional and social factors.

## Introduction

Type 1 diabetes (T1D) is a multifactor disease caused by β-cell destruction (which is mostly immune-mediated) and absolute insulin deficiency. At present, the management of T1D has been improved, but the disease remains incurable. T1D onset is most common in childhood. T1D represents approximately 5–10% of all diabetes diagnoses^1^. Between 70 and 90% of T1D patients at diagnosis exhibit evidence of an immune-mediated process with β-cell autoantibodies. T1D onset is preceded by a preclinical period that lasts approximately 3 years, in which autoantibodies appear in the circulatory system^2^. Immune destruction of the β-cells can be detected by the evaluation of some haematic markers^3^. The disease has strong HLA associations, which explain nearly half of the genetic disease predisposition, while the remainder is due to other genetic polymorphisms^3,4^.

Analysis of genetic disease susceptibility suggests that there is a greater risk of T1D development when the father is affected by the disease than when the mother is affected^5^. On the other hand, there is evidence that a critical role is played by non-genetic factors, including both environmental and host-related factors, which are considered to play decisive roles in the disease process, leading to the manifestation of clinical T1D^6^.

The worldwide incidence of T1D in the age group of 0–15 years varies considerably by region (from 0.5 to 60 per 100,000 children), and the yearly increase ranges from 0.6% to 9.3%. In Europe, the percentage of cases in the age group of 0–15 years will rise by 70%^7^. In the Piedmont region, up to 2013, there were approximately 8,000 cases in this age group with an incidence of 27 new diagnoses per 100,000^8^. Migrant populations tend to show an incidence of diabetes similar to that of most host populations; therefore, a higher T1D incidence in migrant children was observed in Europe^6,9,10^. Such a pronounced increase in incidence cannot be attributable to genetic factors alone. Other major risk factors may include the environment, Western lifestyle and nutrition^10^. Other diseases with immune involvement, such as allergies, exhibit a similar trend, suggesting an inductor role for exogenous factors regarding the increased predisposition to autoimmunity^11^. Preventive measures to reduce the incidence of T1D have not been defined to date. Various factors seem to be involved in modulating the incidence of T1D, including birth delivery mode, feeding, birth weight, infections (especially viral), dietary behaviour, and pharmaceutical use (especially antibiotics). Such factors may contribute to T1D development during the early disease stage^12^; however, compared with genetic factors, environmental factors are less well characterized^13^. β- Cell vulnerability to stress factors has been discussed as the basis of the overload hypothesis^14^. Associations among the microbiome, metabolome, and T1D were shown, highlighting a host-microbiota role in the onset of the disease^12,15^. The origin of the disease process was suspected to be gut microbiota dysbiosis (imbalances in the composition and function of intestinal microbes) associated with altered gut permeability and a major vulnerability of the immune system^6^. Accordingly, evidence obtained from both animal models and human studies suggests that the gut microbiota and the immune system interact closely, emphasizing the role of the intestinal microbiota in the maturation and development of immune functions^16^. Recently, mycobiome-bacteriome interactions, as well as intestinal virome and islet autoimmunity, were hypothesized to be drivers of dysbiosis^17^. Several studies have specifically investigated microbiota composition in children with T1D^18–20^, but the results have not been consistent. Interestingly, most studies are in agreement regarding the reduced microbial diversity observed in subjects with T1D compared with controls; moreover, the microbiota structure in T1D subjects was found to be different from that of control subjects^21,22^. To date, a typical T1D-associated microbiota has not been identified^23–26^. The research also determined that T1D clinical management could be improved by in-depth analysis of the partial remission phase^27^; however, preventive measures are limited and generally focus only on genetic susceptibility^28^ and general population screening for islet autoimmunity^29^. The development of an integrated prediction strategy could be useful for increasing early diagnosis while avoiding onset complications by identifying children at risk of T1D to place under observation and, in the future, to treat with preventive methods^10^.

The aim of this study is to identify environmental, behavioural, and microbial risk factors of T1D onset to develop an integrated T1D preventive management strategy that is suitable for paediatricians in the Piedmont region.

## Results

### Subject description and origin factor analysis

To analyse the origin factor, the study population was subdivided by the children's origins (Italian and migrant, 69 and 27 children, respectively). An analysis of the socio-demographic and behavioural factors examined in the study showed many differences between Italian and migrant children, while other variables appear to be quite homogeneous (Table 1). In the studied cohort, migrant status did not produce a significant increase in T1D onset.

**Table 1:** Summary of the population anthropometric characteristics, comparing cases and controls: number of children involved, sex, age and anthropometrics as the mean and standard deviation.

		Type 1 diabetes patients	Healthy controls
Subjects (number)		40	56
Gender	Male (%)	28 (70.0%)	40 (71.4%)
	Female (%)	12 (30.0%)	16 (28.6%)
Age (years)		8.23 ± 1.42	7.87 ± 1.72
Height (m)		1.33 ± .11	1.30 ± .12
Weight (kg)		29.73 ± 8.06	29.25 ± 9.83
BMI (kg/m^2^)		16.51 ± 2.77	17.01 ± 2.79

Approximately 79% of the children in the cohort had siblings; approximately 40% of the included children lived with a pet in the house, and more than 65% of the children took antibiotics during the first two years of life. The residency zone was notably different between Italians and migrants: the percentage of migrant children living in urban sites was higher but not significant following the adjusted model. Regular sports activities seem to be practised more by Italian children than by migrant children (73.5% vs 51.8%, p = 0.054). A total of 77.9% of Italian children and 55.6% of migrant children were subjected to regular health check-ups (p = 0.017). A significant difference was confirmed for the ages of the migrant mother and father (Table 1), meanly 6 years and 4 years younger respectively at recruitment, respect the Italians (p = 0.017 and p = 0.0425). The analysis of eating habits and nutritional intake revealed that the majority of the children were breastfed. Moreover, the weaning age was 6 months, as recommended. Migrant children showed higher total carbohydrate intake (+ 12%, p = 0.044) and simple carbohydrate intake (+ 24%, p = 0.0045). Moreover, among migrants, the children tended to access food by themselves and to consume meals alone. The percentage of migrant children who ate meals while watching TV was higher but not significant. Finally, the one-course meal was more frequent in migrant families (ratio 1:3, p = 0.006).

The analysis of microbiota and bioindicator species displayed no significant differences between Italian and migrant children: the qRT-PCR measurements showed a trend of greater value for the total bacteria (both for the experimental design with and without probe), *Bacteroides* and *M. smithii* (both using 16S rDNA and *nifH)* in migrant children. The DGGE profile and dendrogram analysis did not show a different clustering pattern based on the origin, and the migrant group showed a trend towards greater α-diversity of the faecal microbiota profiles (Shannon index + 5%). Additionally, the α-diversity analyses in next generation sequencing (NGS) showed a difference in taxonomic units (OTUs), i.e., there were more OTUs in migrants than in Italians, but the difference was not significant, though it was close to the limit of significance (p = 0.057). Furthermore, the phylogenetic diversity index (Faith PD) suggested that the origin of the subjects could influence the structure of the microbial community. Although the overall number of OTUs did not change significantly, the phylogenetic distance of the individual OTUs was greater in the migrant group than in the Italian group, as the OTUs occupied a broader ecological niche in the migrant group.

### T1D risk factors

Previous results indicated that being a migrant child in the Piedmont region is not a significant risk factor for T1D onset^30^. Table 2 shows single logistic regressions performed to estimate the impact of the different variables on the outcome. Notably, the analysis of socio-demographic, behavioural, and nutritional determinants revealed that having parents with at least a high school certificate seems to be a protective factor for T1D onset, even if not significant after adjusted comparisons.

**Table 2:** Oligonucleotide primers, probes and genomic standards used in biomolecular analyses.

Microbial Target	Sequences		Standard Genomic DNA	Ref
*Total Bacteria* *16 s rDNA*	F R	5′ACTCCTACGGGAGGCAGCAG3' 5′ATTACCGCGGCTGCTGG3'	*Desulfovibrio vulgaris* ATCC 29579D-5	^36^
*Total Bacteria* *16 s rDNA*	F R Probe	5′AGAGTTTGATCMTGGCTCAG3’ 5′TTACCGCGGCKGCTGGCAC3’ 5′CCAKACTCCTACGGGAGGCAGCAG3’	*Desulfovibrio vulgaris* ATCC 29579D-5	^36^
*Bacteroidetes* *16 s rDNA*	F R	5′CATGTGGTTTAATTCGATGAT3' 5′AGCTGACGACAACCATGCAG3'	*Bacteroides fragilis* ATCC 25285D-5	^19,38^
*Bacteroides* *16 s rDNA*	F R	5′GAGAGGAAGGTCCCCCAC3' 5′CGCTACTTGGCTGGTTCAG3'	*Bacteroides fragilis* ATCC 25285D-5	^19,38^
*Firmicutes* *16 s rDNA*	F R	5′ATGTGGTTTAATTCGAAGCA3' 5′AGCTGACGACAACCATGCAC3'	*Clostridium acetobutylicum* ATCC 824D-5	^40^
*Bifidobacteria* *16 s rDNA*	F R	5′CTCCTGGAAACGGGTGG3' 5′GGTGTTCTTCCCGATATCTACA3'	*Bifidobacterium longum infantis* ATCC 15697D-5	^39^
*Akkermansia muciniphila* *16 s rDNA*	F R	5′CAGCACGTGAAGGTGGGGAC3' 5′CCTTGCGGTTGGCTTCAGAT3'	*Akkermansia municiphila* ATCC-BAA835D-5	^37^
*M. smithii* *16 s rDNA*	Smit.16S-740 F Smit.16S-862 R Smit.16S FAM	5′CCGGGTATCTAATCCGGTTC-3’ 5′CTCCCAGGGTAGAGGTGAAA3’ 5′CCGTCAGAATCGTTCCAGTCAG3’	*M. smithii* DSM 861	^36^
*M. smithii* *nifH*	Mnif 202 F Mnif 353 R Mnif Probe	5′GAAAGCGGAGGTCCTGAA3' 5′ACTGAAAAACCTCCGCAAAC3' 5′CCGGACGTGGTGTAACAGTAGCTA3'	*M. smithii* DSM 861	^21^
*Bacterial* *16 s rRNA*	357 F-GC 518 R	5′GCclampCTCCTACGGGAGGCAGCAG3' 5′GTATTACCGCGGCTGCTGG3'		^34^
*16 s rDNA V3-V4*	Pro 341 F Pro 805 R	5′TCGTCGGCAGCGTCAGATGTGTATAAGAGACAGCCTACGGGNBGCASCAG3' 5′GTCTCGTGGGCTCGGAGATGTGTATAAGAGCAGGACTACNVGGGTATCTAATCC3'		^41^

High total caloric intake, as well as high protein intake and consumption of total carbohydrates, are associated with only a slightly increased risk of T1D onset.

**Figure 1:**
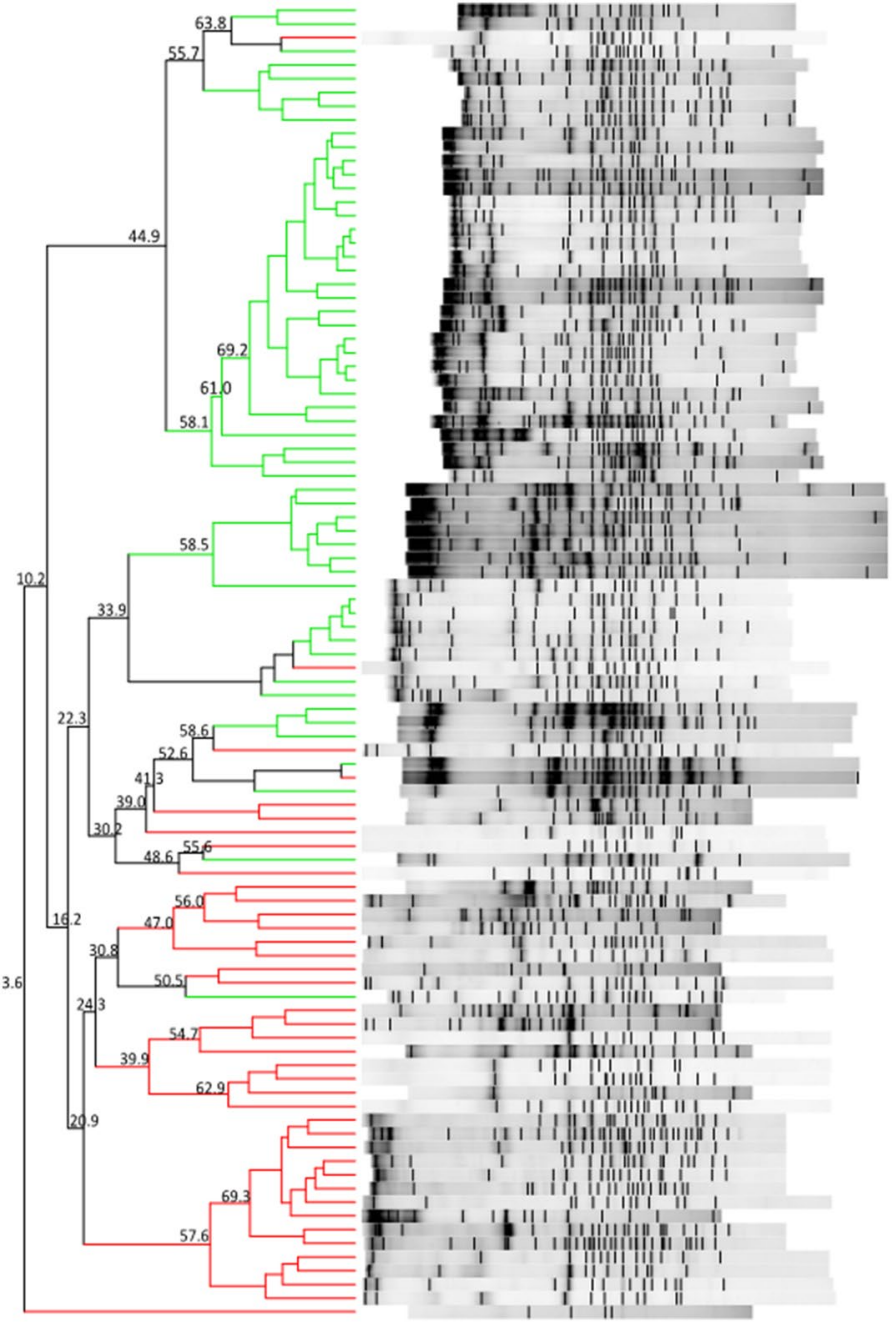
DGGE banding patterns and the results of the analysis in which the Pearson coefficient (numbers reported near the nodes) was used for measuring similarity in banding patterns. The cluster identifies T1D patients (red lines) and healthy children (green lines).

The DGGE gel and the results of the cluster analysis are shown in Fig. 1. The Pearson similarity clustering showed macro beta-diversity differences between the T1D patients and healthy children, with the main division being in two different clusters.

Firmicutes and Bacteroidetes followed by Proteobacteria and Actinobacteria (Table 3) predominantly composed the gut microbiota of all children. In the children with diabetes, an increase in the levels of three members of Bacteroidetes (*Alistipes senegalensis*, *Bacteroides timonensis*, and *Barnesiella intestinihominis*) and three members of Firmicutes (*Christensenella timonensis*,

**Table 3:** Main characteristics of type 1 diabetes patients and healthy children enrolled by subject origin.

		Italians (n. 69)	Migrants (n. 27)	p-value	Adj p-value
**Socio-demographic factors**					
Age at onset		7.85 (± 1.75)	8.46 (± 1.04)	0.093	0.1581
Gender	Female	24 (34.8%)	4 (14.8%)	0.079	0.149
	Male	45 (65.2%)	23 (85.2%)		
Percentile BMI		51.38 (± 32.35)	66.46 (± 36.27)	0.053	0.121
BMI categories	Underweight	1 (1.5%)	2 (7.7%)	**0.016**	0.091
	Normal weight	50 (73.5%)	11 (42.3%)		
	Overweight	11 (16.2%)	7 (26.9%)		
	Obese	6 (8.8%)	6 (23.1%)		
Residency (urban)		43 (63.2%)	23 (85.2%)	**0.048**	0.121
Sport activity		50 (73.5%)	14 (51.8%)	0.054	0.121
The child has siblings		52 (76.5%)	22 (81.5%)	0.785	0.861
The child does regular health check-up		53 (77.9%)	15 (55.6%)	**0.043**	0.121
The child used antibiotics in the first two years of life		46 (67.6%)	17 (63.0%)	0.810	0.861
Breast feeding		57 (83.8%)	24 (88.9%)	0.750	0.861
Weaning age (months)		5.91 (± 2.47)	5.76 (± 1.62)	0.760	0.861
Presence of pets in the house	No pets	14 (24.6%)	7 (28.0%)	0.057	0.121
	Dogs or cats	24 (42.1%)	4 (16.0%)		
	Other pets	19 (33.3%)	14 (56.0%)		
Mother age at recruitment		40.61 (± 4.77)	34.18 (± 5.41)	** < 0.001**	**0.017**
Mother education (at least high school)		50(73.5%)	15 (55.6%)	0.141	0.218
Father age at recruitment		44.19 (± 5.99)	40.29 (± 5.93)	**0.005**	**0.0425**
Father education (at least high school)		38 (55.9%)	17 (63.0%)	0.646	0.861
Important changes in the family contest in the last year		7 (10.3%)	3 (11.1%)	1.000	1.000
**Nutritional anamnesis**					
Total caloric intake (Kcal/die)		1760.23 (± 349.43)	1891.48 (± 372.29)	0.108	0.262
Delta Kcal		-116.15 (± 346.65)	-11.11 (± 366.43)	0.192	0.408
Delta Kcal %		-5.20 (± 17.54)	-0.33 (± 19.06)	0.236	0.446
Total supply of proteins (g)		60.53 (± 13.61)	60.56 (± 13.91)	0.991	1.000
Total supply of lipids (g)		65.40 (± 12.92)	66.52 (± 16.08)	0.724	0.879
Total supply of carbohydrates (g)		232.51 (± 58.51)	259.70 (± 59.09)	**0.044**	0.1496
Total supply of CHO RA (g)		71.02 (± 26.39)	88.85 (± 28.45)	**0.0045**	**0.0255**
The child has access to food by himself when he/she is at home		39 (57.4%)	23 (85.2%)	**0.016**	0.0544
The child consumes meals alone	Always alone	3 (4.4%)	12 (44.4%)	** < 0.001**	**0.006**
	Always with an adult	61 (89.7%)	16 (37.1%)		
	Both	4 (5.9%)	5 (18.5%)		
Number of extra meals a day	0	1 (1.5%)	0 (0%)	0.330	0.4675
	1	2 (2.9%)	0 (0%)		
	2	36 (52.9%)	10 (37.1%)		
	3	21 (30.9%)	10 (37.1%)		
	4	8 (11.8%)	7 (25.8%)		
The child consumes meals while watching TV		43 (63.2%)	22 (81.5%)	0.094	0.262
The child consumes sweets more than three times a week		39 (57.3%)	16 (59.3%)	1.000	1.000
Child family consumes meals all together		62 (91.2%)	22 (81.5%)	0.284	0.4675
Family talks during the meal		61 (89.7%)	22 (81.5%)	0.312	0.4675
The child often asks for supplementary portions of food		35 (51.5%)	16 (59.2%)	0.649	0.849
The main meal of child	Lunch	14 (20.9%)	7 (25.9%)	0.892	1.000
	Dinner	51 (76.1%)	20 (74.1%)		
	Both	2 (2.99%)	0 (0%)		
Meals	One course meals	11 (16.2%)	14 (51.9%)	**0.001**	**0.006**
	Not one course meals	54 (79.4%)	12 (44.4%)		
	Both	3(4.4%)	1 (3.7%)		
**Microbiota**					
*Akkermansia muciniphila* (Log gene copies/g stool)		6.21 (± 1.29)	6.66 (± 1.44)	0.1475	0.228
*Bacteroides spp.* (Log gene copies/g stool)		8.56 (± 0.91)	9.08 (± 0.74)	**0.0092**	0.060
Bacteroidetes (Log gene copies/g stool)		8.38 (± 1.31)	8.81 (± 0.83)	0.124	0.228
Total bacteria Probe (Log gene copies/g stool)		9.48 (± 0.96)	9.96 (± 0.74)	**0.019**	0.062
Total bacteria SYBR (Log gene copies/g stool)		9.95 (± 0.63)	10.27 (± 0.63)	**0.025**	0.065
Firmicutes (Log gene copies/g stool)		10.38 (± 0.77)	10.59 (± 0.87)	0.259	0.306
*Bifidobacterium spp.* (Log gene copies/g stool)		6.89 (± 1.16)	7.02 (± 0.97)	0.597	0.597
*Methanobrevibacter smithii 16S* (Log gene copies/g stool)		5.24 (± 1.22)	6.15 (± 1.74)	**0.004**	0.052
*Methanobrevibacter smithii Nihf* (Log gene copies/g stool)		5.16 (± 1.05)	5.83 (± 1.53)	**0.0151**	0.062
Simpson index		0.11 (± 0.04)	0.10 (± 0.05)	0.1580	0.228
Shannon index		2.45 (± 0.29)	2.58 (± 0.31)	**0.0402**	0.087
Firmicutes/Bacteroidetes ratio		1.29 (± 0.37)	1.22 (± 0.17)	0.3224	0.349
Margalef index		2.53 (± 0.68)	2.72 (± 0.65)	0.2145	0.279

The continuous variables are expressed as means and standard deviations; the categorical variables are expressed as absolute numbers and percentages. Adj p-value: adjusted for multiple comparisons.

*Ruminococcus bromii*, and *Urmitella timonensis*) was observed by sequencing.

Furthermore, other notable results were obtained by NGS analyses. The taxonomic analysis revealed that the gut microbiota of the study participants was composed of nine relevant phyla: Firmicutes, Bacteroidetes, Actinobacteria, Proteobacteria, Verrucomicrobia, Euryarchaeota, Tenericutes, Cyanobacteria, and an unclassified phylum.

Moreover, beta-diversity analyses were carried out to highlight the differences among the samples based on the structures of their microbial communities. The weighted UniFrac metric showed that the samples were not subdivided into clusters. The intragroup and intergroup distances were comparable, and there was no separation between the clusters. These findings were confirmed by the Permanova test. Finally, analyses of the differential abundance were performed to compare the increase or decrease in the abundance of one or more bacteria in the case and control groups. DeSeq2 showed 48 significantly abundant OTUs (p < 0.001). The most abundant OTU was *Rikenellaceae* followed by *Prevotellaceae* (*Prevotella copri*), *Barnesiellaceae*, *Lachnospiraceae,* and *Ruminococcaceae* (*Ruminococcus bromii*), which were significantly more abundant in children with diabetes.

**Figure 2:**
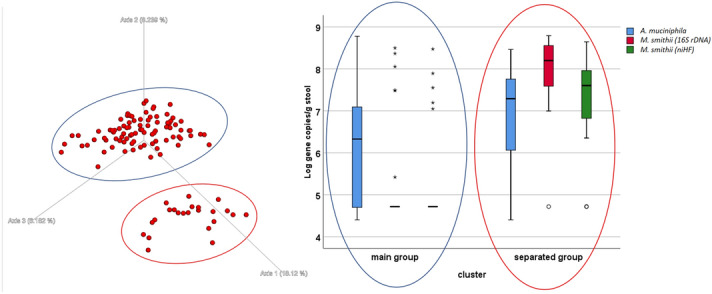
Left-Unweighted UniFrac graph of the NGS results. There are two identifiable groups: the blue circle (main group) and the red circle (separated group). No experimental hypothesis was confirmed for the cluster definition. On the Right: box plot of the qRT-PCR results for some microbiological targets (*Akkermansia muciniphila* and *Methanobrevibacter smithii*), the difference between the groups is significant (t-test p < 0.05).

The difference in the results observed between methods is an interesting discussion point. The methods are characterized by different sensitivities; they represent different molecular perspectives regarding the faecal microbiota. When a method with a higher sensibility is used (NGS), a flattening effect is possible. On the other hand, the major abundance of such genera as *Ruminococcu*s was confirmed by different microbiota study methods, which is in keeping with the qRT-PCR results. A group of 23 samples showed different clusterization compared to the others (Fig. 2, left). This small group was not different from the main group regarding any characteristics. The only significant difference was observed for the *M. smithii* presence and the *A. muciniphila* levels, both of which were higher in the separated group (Fig. 2, right). *A. muciniphila* was proposed as a probiotic^31^, while *M. smithii* has been characterized as the most abundant methanogen in the gut^32^.

The qRT-PCR gut microbiota analysis indicated significant differences among T1D patients and healthy children (Table 2). The logistic regression analysis showed that the increase in the Margalef index was associated with a decrease in the likelihood of disease onset (OR 0.20; 95% CI 0.09–0.46, p = 0.000). Increased Firmicutes levels and decreased Bacteroidetes levels were significant risk factors for T1D (OR 7.49; 95% CI 3.25–17.28, p = 0.0001; OR 0.28; 95% CI 0.15–0.51 p = 0.0001, respectively). Moreover, *Bifidobacterium spp.* was a protective factor for T1D onset (OR 0.20; 95% CI 0.10–0.38, p = 0.0001).

The multivariable analysis produced a R^2^ = 0.6259 (p < 0.001). After adjusting for confounding factors, the likelihood of having diabetes is significantly higher in those with higher amount of Firmicutes, lower amount of *Bifidobacterium spp* and a higher amount of total carbohydrate intake (Table 4).

**Table 4:** Logistic regressions: likelihood of having diabetes.

		Likelihood of having diabetes			
		OR	95% IC	p-value	Adj p-value
**Socio-demographic factors**					
Age at recruitment		1.15	0.89—1.49	0.290	0.6195
Gender (female)		1.07	0.43—2.61	0.879	0.925
Percentile BMI		0.99	0.98—1.003	0.179	0.507
BMI categories	Underweight	0.39	0.034—4.61	0.461	0.7785
	Overweight	0.25	0.02—3.34	0.295	0.6195
	Obese	0.36	0.02—5.11	0.448	0.779
Residency (rural)		0.96	0.39—2.32	0.924	0.925
The child consumes meals at school		0.51	0.19—1.40	0.193	0.507
The child consumes meals at home more than two times a week		0.42	0.17—1.07	0.070	0.2555
Sport activity		1.23	0.51—2.95	0.641	0.785
Having siblings		0.75	0.28—1.99	0.563	0.785
Having done regular health check-up		0.37	0.15—0.93	**0.036**	0.252
Use of antibiotics in the first two years of life		0.74	0.31—1.75	0.503	0.7785
Breastfeeding		0.68	0.22—2.14	0.519	0.7785
Weaning age (months)		0.68	0.46—1.03	0.068	0.2555
Presence of dogs and/or cats in the house		0.95	0.37—2.44	0.925	0.925
Mother age at recruitment		0.98	0.91—1.06	0.673	0.785
Mother education (at least high school)		0.34	0.14—0.83	**0.018**	0.189
Father age at recruitment		1.01	0.95—1.08	0.671	0.785
Father education (at least high school)		0.33	0.14—0.77	**0.011**	0.189
Important changes in the family contest in the last year		3.68	0.88—15.22	0.073	0.2555
**Nutritional anamnesis**					
Total caloric intake (Kcal/die)		1.0023	1.0009—1.0036	**0.001**	**0.005**
Total supply of proteins (g)		1.06	1.02—1.10	**0.002**	**0.007**
Total supply of lipids (g)		1.03	1.002—1.069	**0.036**	0.072
Total supply of carbohydrates (g)		1.01	1.005—1.022	**0.001**	**0.005**
Total supply of CHO RA (g)		1.03	1.007—1.045	**0.006**	**0.015**
The child consumes more than two extra meals		1.67	0.73—3.82	0.224	0.329
The child consumes meals while watching TV		0.58	0.24—1.40	0.230	0.329
The child consumes sweets more than three times a week		1.08	0.47—2.47	0.859	0.859
Child family consumes meals all together		0.54	0.15—1.91	0.339	0.4238
The child often asks for supplementary portions of food		1.44	0.62—3.28	0.389	0.432
**Microbiota**					
*Akkermansia muciniphila* (Log gene copies/g stool)		0.84	0.62—1.14	0.260	0.423
*Bacteroides spp.* (Log gene copies/g stool)		0.79	0.50—1.25	0.317	0.443
Bacteroidetes (Log gene copies/g stool)		0.28	0.15—0.51	**0.000**	**0.000**
Total bacteria Probe (Log gene copies/g stool)		0.72	0.46—1.12	0.147	0.318
Total bacteria SYBR (Log gene copies/g stool)		0.69	0.37—1.31	0.259	0.423
Firmicutes (Log gene copies/g stool)		7.49	3.25—17.28	**0.000**	**0.000**
*Bifidobacterium spp.* (Log gene copies/g stool)		0.20	0.10—0.38	**0.000**	**0.000**
*Methanobrevibacter smithii 16S* (Log gene copies/g stool)		0.97	0.73—1.30	0.858	0.930
*Methanobrevibacter smithii Nihf* (Log gene copies/g stool)		1.04	0.75—1.44	0.824	0.930
Simpson index		1.14	0.00005—23,610.2	0.980	0.980
Shannon index		0.51	0.13—2.03	0.341	0.443
Firmicutes/Bacteroidetes ratio		34,288.2	637.20—1,845,077	**0.000**	**0.000**
Margalef index		0.20	0.09—0.46	**0.000**	**0.000**

The continuous variables are shown on a light grey background; the categorical variables are shown on a white background. Adj p-value: adjusted for multiple comparisons. Significant p-values are bolded.

## Discussion

T1D is an important disease that affects health with onset primarily occurring in childhood. At present, there is no cure for this disease, and only disease management is possible. The disease burden of T1D is immense, especially considering the number of years of life lost due to disability but also the years of life lost due to premature death. The life expectancy for T1D patients is approximately 16 years shorter than that of the comparable healthy population^33^. Even if relevant risk factors are known, to date, such scientific determinants do not include a screening programme for preventive purposes. Of course, preventive action must be considered as a systematic process that focuses on the main risk factors to identify children at higher risk of T1D and to suggest efficacious preventive treatments. In the study, the main T1D onset risk factors seem to be identifiable in the composition of the microbiota and, in particular, the microbiota α-diversity, Firmicutes and Bacteroidetes levels and their ratio, as well as the *Bifidobacterium* level. Similar evidence was obtained by other studies, which observed both higher Bacteroidetes in T1D patients^34,35^ and less abundant anti-inflammatory genera in children with multiple islet autoantibodies^36^. Reduced microbial diversity appears to become significant between seroconversion and overt T1D^15^. A significant difference in the *Bifidobacterium* level was observed in different studies, including both a small cohort of autoimmune children^37,38^ and a larger population associated with such protective factors as breastfeeding^21^. At the genus level, a significant difference in, for example, *Blautia* (increased in patients), was observed^39^; however, in other studies, different single species (*Bacteroides ovatus*) seem to be more abundant in patients than in the controls^18^. However, prior studies suggest the presence of duodenal mucosa abnormalities in the inflammatory profile for T1D patients^22,40^ and on the T1D-related changes in the gut microbiota, even if proving the causality of these factors has remained challenging^21^.

The characterization of the microbiota is rapidly evolving. Traditional methods that are not as sensitive as PCR-DGGE are still suitable, while NGS methods are expanding. Sophisticated whole-genome sequencing methods integrated with metabolomics and proteomics have been proposed. However, the large amount of data, being affected by multiple confounding factors, has not had a clear impact on T1D prevention strategies. The development of a simple method to describe microbiota modulation using validated biomarkers, which could serve as a rapid screening test, may be warranted.

Another risk factor is the occurrence of stress due to a traumatic or emotional experience. This stress seems to be able to affect the autoimmunity process. Therefore, particular attention could be paid to such risk factors for T1D risk in children.

A high education level of one or both parents could be also protective, suggesting that socioeconomic factors affect the T1D risk. Other factors, identified as significant risk modulators among behavioural and nutritional factors, had minor effects.

The study has some potential limitations, including susceptibility to bias in recollection about exposure and reverse causality. The exposure recollection could be biased, but this issue can be less influential at the onset, as in this study. Moreover, recruitment at the onset guarantees a temporal coherence of the exposure with respect to the disease onset.

T1D is one of the most frequently diagnosed diseases in children; however, it is not a high-incidence disease. The prospective inclusion of a large number of healthy children, which is needed for the observation of enough cases, requires a very long time of observation. Moreover, a restricted age range was necessary in children for the rapid changes in behaviour and microbiota. This requirement resulted in an additional included subject restriction. On the other hand, the study of multifactorial diseases with poorly understood pathogenic pathways is imperative, even if it is at risk for obtaining less conclusive evidence. Of course, such a study alone could not elucidate the causation process, but the evidence obtained could be important for the selection of higher-risk subpopulations, planning of future research, and improving prevention.

Identification of a higher-risk subpopulation is strictly relevant for the subsequent validation of an efficient preventive screening to be produced with a prospective method. Of course, the pathogenesis of type 1 diabetes has not been fully elucidated to date; however, in this study, various factors (associated with both the disease and the microbiota composition) were included, such as the origin of the children, the age of the mother, the age of breastfeeding and the age of weaning. Other possible confounding factors not included in our analysis are viral infections, particularly enteroviruses, and preterm birth; however, there was no clear consensus regarding these novel factors at the beginning of the study.

Concerning the microbiota, the knowledge is still incomplete, and various factors can interact to produce a T1D risk modulation that is not explainable at present. Moreover, the results obtained using different techniques were also dissimilar (for example, clusterization due to β-diversity analysis). This finding is likely due to the different sensitivities of the applied methods^41^. Furthermore, even if the time between the symptom comparison and the diagnosis is very short, there is a danger of biased estimates due to reverse causality.

In conclusion, this study confirmed that T1D onset risk is modulated by compositional changes in the gut microbiota and that such evidence must be employed to devise preventive measure. The results showed that the gut microbial indicators found in children with T1D differ from those found in healthy children. These findings also pave the way for new research attempting to develop strategies to control T1D development by modifying the gut microbiota. However, a better knowledge of gut microbial composition associated with the development of T1D must be obtained to choose the best treatment^10,42–45^.

In brief, direct or indirect manipulations of the intestinal microbiome may provide effective measures for preventing or delaying the disease process leading to the manifestation of clinical T1D. At present, a preventive strategy could be developed that includes the main genetic and microbiome risk factors. Then, this strategy could be applied to healthy children to reduce the burden of T1D.

## Methods

### Study design and participants

The case–control study began in January 2016^46^ and ended in September 2018 (case–control phase of clinicaltrial.gov Protocol ID: G12114000080001). The work was conducted following the STROBE Statement for a case–control study. The activity is bicentric and includes the two main paediatric hospitals in the Piedmont region (located in Torino and Novara), which cover the clinical management for cases of T1D in the region. The ethics committees of the two hospitals approved the research activities during 2015 (“Comitato etico interaziendale A.O.U. Ordine Mauriziano di Torino ASLTO1” with record number 0117120 and “Comitato etico Interaziendale A.O.U. “Maggiore della Carità” ASL BI, NO, VCO” record number 631/CE).

The recruitment included 40 paediatric patients with T1D (cases) and 56 healthy children (controls), who were comparable in terms of age, gender, and ethnicity to avoid bias. The included subjects represent the most convenient sample possible. The inclusion criteria were age (5–10 years), normal weight, and residence in Piedmont. Exclusion criteria were celiac disease, chronic disease diagnosis, eating disorders, active infections, use of antibiotics and/or probiotics and/or any other medical treatment that influences intestinal microbiota during the 3 months before recruitment and children with parents of mixed origins (Italian and migrant) for the exclusion of important confounding factors due to genetic and cultural mixed backgrounds^19^.

The T1D children were integrated into the study at disease onset, with hyperglycaemia, with or without ketoacidosis, polyuria symptoms, a high value of glycated haemoglobin (HbA1c > 42 mmol/mol) and T1D-specific autoantibody positivity. Healthy children were contacted by paediatricians in the territory of the acute care system. The guardians of the enlisting children read, understood, and then signed informed consent forms following the declaration of Helsinki. A module is prepared for parents, children, and mature children^47^. All the following methods were carried out following relevant guidelines and regulations when available. A questionnaire was given to the parents containing items and questions to retrieve data on the family contest with particular regards to emotive stressors, such as mourning or separation, anthropometrics, and socio-demographic, nutritional, and behavioural information.

Anthropometric and nutritional data included weight, height, body mass index (BMI), food frequency based on 24-h recall and a food frequency questionnaire (FFQ), neonatal feeding, and age of weaning. The anthropometric parameters (weight and height) were measured according to standard recommendations. The BMI values were interpreted according to the WHO criterion. The 24-h recall technique reconstructed the meals and food intake on a recent "typical" day, estimating the bromatological inputs according to a food composition database for epidemiological studies in Italy (BDA). The FFQ, developed for the study, focused on the consumption of certain food categories (those containing sugars, fibre, omega-3, calcium, vitamin D, condiments, and cereals) and eating habits (e.g., alone or with adults, in front of the TV).

Twenty-eight percent of the involved population is migrants (both parents not Italian). Such data are consistent with the percentage of newborns from non-Italian mothers, which is approximately 30% in northern Italy^48^. The migrant group included children coming mainly from northern Africa and Eastern Europe. The migration involved the parents and sometimes the children; on average, the included children as migrants were residents in Italy for less than 5 years. At the end of recruitment, no significant differences were observed between the case and control groups for age, sex composition, and origins (criteria for pairing) or for height, weight, and BMI (T-test, p > 0.05) (Table 5).

**Table 5:** Bacterial species identified by sequencing of the most representative DGGE bands amplified from fecal DNA of type 1 diabetes (cases) and healthy children (controls).

Closet Relative	Identity	Phylum	Class	Order	Family	Genus	Cases	Controls
*Alistipes putredinis*	100%	Bacteroidetes	Bacteroidia	Bacteroidales	Rikenellaceae	Alistipes	10% (4)	35.7% (20)
*Alistipes senegalensis*	96%	Bacteroidetes	Bacteroidia	Bacteroidales	Rikenellaceae	Alistipes	75% (30)	50% (28)
*Bacteroides coprocola*	98%	Bacteroidetes	Bacteroidia	Bacteroidales	Bacteroidaceae	Bacteroides	5% (2)	0% (0)
*Bacteroides dorei*	100%	Bacteroidetes	Bacteroidia	Bacteroidales	Bacteroidaceae	Bacteroides	67.5%(27)	80.3% (45)
*Bacteroides faecis*	99%	Bacteroidetes	Bacteroidia	Bacteroidales	Bacteroidaceae	Bacteroides	15% (6)	16.1% (9)
*Bacteroides finegoldii*	94%	Bacteroidetes	Bacteroidia	Bacteroidales	Bacteroidaceae	Bacteroides	17.5% (7)	46.4% (26)
*Bacteroides intestinalis*	97%	Bacteroidetes	Bacteroidia	Bacteroidales	Bacteroidaceae	Bacteroides	90% (36)	92.8% (52)
*Bacteroides timonensis*	100%	Bacteroidetes	Bacteroidia	Bacteroidales	Bacteroidaceae	Bacteroides	20% (8)	10.7% (6)
*Barnesiella intestinihominis*	99%	Bacteroidetes	Bacteroidia	Bacteroidales	Barnesiellaceae	Barnesiella	72.5% (29)	66.1% (37)
*Bifidobacterium faecale*	100%	Actinobacteria	Bifidobacteriales	Bifidobacteriaceae	Bifidobacterium		0% (0)	28.6% (16)
*Christensenella timonensis*	93%	Firmicutes	Clostridia	Clostridiales	Christensenellaceae	Christensenella	40% (16)	16.1% (9)
*Clostridium dakarense*	97%	Firmicutes	Clostridia	Clostridiales	Peptostreptococcaceae	Romboutsia	5% (2)	5.3% (3)
*Colidextribacter massiliensis*	93%	Firmicutes	Clostridia	Clostridiales	Colidextribacter		42.5% (17)	39.3% (22)
*Coprobacter fastidiosus*	82%	Bacteroidetes	Bacteroidia	Bacteroidales	Barnesiellaceae	Coprobacter	0% (0)	5.3% (3)
*Dialister propionicifaciens*	89%	Firmicutes	Negativicutes	Veillonellales	Veillonellaceae	Dialister	10% (4)	25% (14)
*Dialister succinatiphilus*	100%	Firmicutes	Negativicutes	Veillonellales	Veillonellaceae	Dialister	5% (2)	30.3% (17)
*Escherichia coli*	99%	Proteobacteria	Gammaproteobacteria	Enterobacterales	Enterobacteriaceae	Escherichia	65% (26)	76.8% (43)
*Eubacterium rectale*	100%	Firmicutes	Clostridia	Clostridiales	Lachnospiraceae		3 5% (14)	33.9% (19)
*Fusicatenibacter saccharivorans*	100%	Firmicutes	Clostridia	Clostridiales	Lachnospiraceae	Fusicatenibacter	87.5% (35)	100% (56)
*Megasphaera massiliensis*	99%	Firmicutes	Negativicutes	Veillonellales	Veillonellaceae	Megasphaera	37.5% (15)	41.1% (23)
*Negativibacillus massiliensis*	91%	Firmicutes	Clostridia	Clostridiales	Ruminococcaceae	Negativibacillus	20% (8)	26.8% (15)
*Parabacteroides johnsonii*	95%	Bacteroidetes	Bacteroidia	Bacteroidales	Tannerellaceae	Parabacteroides	10% (4)	23.2% (13)
*Prevotella copri*	99%	Bacteroidetes	Bacteroidia	Bacteroidales	Prevotellaceae	Prevotella	57.5% (23)	76.8% (43)
*Pseudoflavonifractor phocaeensis*	94%	Firmicutes	Clostridia	Clostridiales	Pseudoflavonifractor		20% (8)	51.8% (29)
*Romboutsia timonensis*	100%	Firmicutes	Clostridia	Clostridiales	Peptostreptococcaceae	Romboutsia	82.5% (33)	87.5% (49)
*Roseburia faecis*	99%	Firmicutes	Clostridia	Clostridiales	Lachnospiraceae	Roseburia	60% (24)	83.9% (47)
*Ruminococcus bromii*	93%	Firmicutes	Clostridia	Clostridiales	Ruminococcaceae	Ruminococcus	45% (18)	41.1% (23)
*Subdoligranulum variabile*	97%	Firmicutes	Clostridia	Clostridiales	Ruminococcaceae	Subdoligranulum	57.5% (23)	78.6% (44)
*Urmitella timonensis*	88%	Firmicutes	Tissierellia	Tissierellales	Tissierellaceae	Urmitella	40% (16)	39.3% (22)

### Sample collection and DNA extraction

A kit for stool collection was delivered to each study participant following a validated procedure^49,50^ and using a Fecotainer device (Tag Hemi VOF, Netherlands). Faecal samples were homogenized within 24 h in the laboratory, and five 2 g aliquots were stored at − 80 °C until DNA isolation was performed. Total DNA extractions from the stool samples were performed using the QiaAmp PowerFecal DNA Kit (QIAGEN, Hilden, Germany). The nucleic acids were quantified using a NanoQuant Plate (TECAN Trading AG, Switzerland), which allows quantification using a spectrophotometer read at 260 nm. The spectrophotometer used was the TECAN Infinite 200 PRO, and the software was i-Control (version 1.11.10). The extracted DNA concentrations ranged from 1.1–155.5 ng/μl (mean 41.35 ± 38.70 ng/μL). Samples were stored at –20 °C until molecular analysis was performed.

### PCR-DGGE

The PCR products for denaturing gradient gel electrophoresis (DGGE) were obtained by amplifying the bacterial 16S rRNA genes following a marker gene analysis approach^51^. The primer pairs were 357F-GC and 518R (Table 6)^52^. All PCRs were performed with the T100 Bio-Rad Thermocycler in a 25-μl reaction volume containing 1X Master Mix (166–5009, Bio-Rad, Berkeley, CA, USA), 0.02 bovine serum albumin (BSA), 0.4 μM of each primer, and 2 μl of DNA diluted 1:10 in sterile DNase-treated water. DGGE was carried out using a DCode System (Bio-Rad) with a 30–50% denaturing gradient of formamide and urea^53^. Electrophoresis ran at 200 V for 5 h at 60 °C in 1X TAE buffer. Gels were stained for 30 min with SYBR Green I nucleic acid gel stain (10.000X in DMSO, S9430, Sigma-Aldrich, USA) and were visualized using the D-Code XR apparatus from Bio-Rad. Then, DGGE bands were excised, incubated overnight at − 20 °C, washed, and crushed in 20 μl of molecular-grade water. The supernatant (2 μl) was used as a template and reamplified, as previously described, without BSA and using modified linker-PCR bacterial primers (357F-GC; 518R-AT-M13) (Table 6) ^19,52,54–60^. The obtained PCR products were sequenced with Sanger sequencing (Genechron-Ylichron S.r.l.). The sequence similarities were obtained by the National Centre for Biotechnology Information (NCBI) database using nucleotide Basic Local Alignment Search Tool (BLASTn) analysis.

**Table 6:** Multivariable logistic regression model assessing potential risk factors of T1D.

	OR*	95% CI**
Total charbohydrate intake (g)	1.03	1.01—1.05
Firmicutes (Log gene copies/g stool)	7.30	2.26—23.54
*Bifidobacterium spp* (Log gene copies/g stool)	0.13	0.05—0.34

*Odds Ratio, adjusted also for age and gender.

**Confidence Interval.

### NGS

High-throughput DNA sequencing and analysis were conducted by BMR Genomics s s.r.l. The V3-V4 region of 16S rDNA was amplified using the MiSeq 300PEPro341F and Pro805R primer pair^6^. The sample reads were above 12*10^6^. The reaction mixture (25 μl) contained 3–10 ng/μl genomic DNA, Taq Platinum HiFi (Invitrogen, Carlsbad, CA), and 10 μM of each primer. The PCR conditions for amplification of DNA were as follows: 94 °C for 1 min (1X), 94 °C for 30 s, 55 °C for 30 s, 68 °C for 45 s (25X), and 68 °C for 7 min (1X). PCR products were purified through Agencourt XP 0.8X Magnetic Beads and amplified shortly with the Index Nextera XT. The amplicons were normalized with SequalPrep (Thermo Fisher) and multiplexed. The pool was purified with Agencourt XP 1X Magnetic Beads, loaded onto MiSeq, and sequenced with the V3 chemistry-300PE strategy.

### qRT-PCR

Starting from the extracted DNA, the following microbial targets were quantified by qRT-PCR using a CFX Touch Real-Time PCR Detection System (Bio-Rad-Hercules, CA) and CFX Manager (3.1 Software): total Bacteria, Bacteroidetes, *Bacteroides* spp., Firmicutes, *Bifidobacterium* spp., *Akkermansia muciniphila,* and *Methanobrevibacter smithii*. Total bacteria and *M. smithii* were detected following two reaction designs. For *M. smithii*, the analysis was performed using as target both the *16S rDNA* and then a specific functional gene (*nifH*). For total bacteria, quantification was carried out using a protocol with or without a probe. For the determination of total bacteria (method without probe), Bacteroidetes, *Bacteroides* spp., Firmicutes, *Bifidobacterium* spp. and *Akkermansia muciniphila*, 2 µl of 1:10 extracted DNA was added to a reaction mixture consisting of 10 µl Sso Advance SYBR Green Supermix (172–5261, Bio-Rad), 0.5 µl each of the forward and reverse primers (10 µM final concentration) and 7 µl of ultrapure water in a 20 µl final reaction volume. The reaction conditions were set as follows: 95 °C for 3 min (1X), 95 °C for 10 s, and 59 °C for 15 s (57 °C for Bacteroidetes spp. and 60 °C for Firmicutes), 72 °C for 10 s (39X), 65 °C for 31 s, 65 °C for 5 s + 0.5 °C/cycle, ramp 0.5 °C/s (60X). Moreover, for the determinations of *M. smithii* and total bacteria (method with probe), the reaction was as follows. Two microlitres of 1:10 extracted DNA was added to a reaction mixture consisting of 10 µl IQ Multiplex PowerMix (Bio-Rad-Hercules, CA), 0.2 µl of the molecular probe (10 µM), 0.5 µl each of the forward and reverse primers (10 µM final concentration) and 6.8 µl of ultrapure water in a 20 µl final reaction volume. The reaction conditions were 95 °C for 3 min (1X), 95 °C for 10 s, 59 °C for 15 s, 72 °C for 15 s (39X), and 72 °C for 5 min. Standard curves were produced with serial six-fold dilutions of genomic DNA from the microorganism target, provided by ATCC (Manassas, Virginia, USA) or DSMZ (Braunschweig, Germany). All PCR tests were carried out in triplicate. Table 6 provides detailed information regarding oligonucleotide sequences and genomic standards^19,54–60^. The PCR efficiencies were always between 90 and 110%. To confirm the amplification of each target, gel electrophoresis was performed on 2% agarose gels.

### Data elaboration and statistical analyses

The statistical analysis was performed using STATA version 11.0. Moreover, the data on the included T1D patients and healthy controls were elaborated to highlight the likelihood of having diabetes. A descriptive analysis of the variables was conducted. The data were reported as absolute numbers and percentages for categorical variables and as means and standard deviations for continuous variables. Moreover, the subjects were divided by individual origins into two groups: Italian and migrant, considering the origin of the children and their families, to show differences in the distribution of disease determinants and to assess whether being a migrant could be associated with T1D onset. Differences between Italian and migrant children were assessed using the χ^2^ test with Fisher’s correction for categorical variables and Student’s t-test for continuous variables. Univariable logistic regression was then performed to estimate the impact of sociodemographic, nutritional, and microbiota-related variables on the outcome. These associations were expressed as odds ratios (OR) at a 95% confidence interval (CI). Moreover, the adjusted p-value for multiple comparisons was calculated using the Benjamini and Hochberg false discovery rate method. We conducted multivariable analyses including various variables (age, gender, Firmicutes, *Bifidobacterium spp*., and total carbohydrate intake) and the risk of type 1 diabetes using logistic regression models. The Spearman rank-order correlation coefficient was also determined to assess the relationships between variables. A p-value p < 0.05 was considered significant for all analyses.

The DGGE gel analysis was performed with Bionumerics 7.2. The hierarchical classification was performed with a UPGMA system (1% tolerance and optimization level) and Pearson correlation. Simpson's diversity index, Shannon’s index, and Margalef index were calculated for each DGGE profile to evaluate alpha diversity.

NGS bioinformatics analysis was performed with the software pipeline Qiime2. The reads were cleaned up by the primers using the software Cutadapt (version 2018.8.0) and processed with the software DADA2. The sequences were trimmed at the 3′ end (forward: 270 bp; reverse 260 bp), filtered by quality, and merged with default values. Subsequently, the sequences were elaborated to obtain unique sequences. In this phase, the chimaeras (denoised-paired) are also eliminated. The sequences were clustered against unique sequences at 99% similarity. The taxonomies of both GreenGenes (version 13–8) and Silva (version 132) were assigned to the OTU sequences. Alpha-diversity analyses were performed on all samples using the observed OTUs, Shannon, Pielou's evenness, and Faith PD indices, and for each index, the Kruskal–Wallis test was used to verify the significance of the comparisons between samples. Beta-diversity analyses were performed on all samples using the Bray–Curtis, Jaccard, and UniFrac metrics (weighted and unweighted). Multivariable statistical analyses were performed using the PERMANOVA, Adonis, and ANOSIM tests; instead, the analysis of the differential abundance was based on the packages of R (MetagenomeSeq, DeSeq2, and ANCOM).

## Data Availability

The database includes human data that are available upon reasonable request.
